# Subjective social status and health of Saudi medical students: a cross-sectional study on the role of peer comparisons

**DOI:** 10.1080/07853890.2026.2708520

**Published:** 2026-08-03

**Authors:** Armen A. Torchyan, Abdulrahman H. Alsergani, Faisal A. Alhejji, Fahad A. Aldhafian, Abdulrahman A. Almuhaideb, Abdullah A. Alenazi, Nizar A. Alfiaar, Satenik L. Papikyan, Leena R. Baghdadi, Inge Houkes, Hans Bosma

**Affiliations:** a Department of Social Medicine, Care and Public Health Research Institute (CAPHRI), Faculty of Health, Medicine and Life Sciences, Maastricht University, Maastricht, the Netherlands; b College of Medicine, King Saud University, Riyadh, Saudi Arabia; c Department of Family and Community Medicine, College of Medicine, King Saud University, Riyadh, Saudi Arabia

**Keywords:** Medical students, mental health, physical health, subjective social status, Saudi Arabia

## Abstract

**Background:**

This study examined the relationship between peer subjective social status (SSS), a measure of perceived social position within social hierarchies, and health outcomes in a young adult population.

**Methods:**

A cross-sectional study was conducted in 2025 at the College of Medicine, King Saud University, Saudi Arabia, involving 390 undergraduate students aged 18 and older. Peer SSS was assessed using the validated MacArthur Scale, a 10-rung ladder representing perceived social standing relative to peers. Multiple logistic regression models were used to estimate associations between peer SSS and four health outcomes, adjusting for age, sex, and indicators of parental socioeconomic position.

**Results:**

Each one-rung increase in peer SSS was associated with 15–33% lower odds of poor health outcomes, including less than good physical health (odds ratio [OR] = 0.85, 95% confidence interval [CI] = 0.72–1.00), less than good mental health (OR = 0.81, 95% CI = 0.68–0.95), low psychological well-being (OR = 0.81, 95% CI = 0.69–0.95), and low life satisfaction (OR = 0.67, 95% CI = 0.54–0.82). Sensitivity analyses showed no effect modification by sex, age, or parental socioeconomic position.

**Conclusion:**

These findings suggest that perceived social position within peer hierarchies is an important correlate of physical and mental health in young adults, independent of family background. Further longitudinal and qualitative research is needed to clarify the direction of the association and the underlying mechanisms, and to examine whether addressing the psychosocial consequences of status comparisons may be relevant for reducing health inequalities in competitive social environments.

**Key messages**
- Perceived peer social status is strongly associated with both physical and mental health among medical students, independent of parental socioeconomic background.
- Students with lower peer status have substantially higher odds of poor health outcomes, including reduced well-being and life satisfaction.
- Competitive peer dynamics within medical education may be an important factor related to student well-being and warrant further investigation.

## Introduction

In many countries, medical students are frequently exposed to elevated levels of stress, which can substantially affect their mental health and overall well-being [1,2], and recent evidence indicates that psychological distress remains common among health sciences students, including those in Saudi Arabia and other medical science settings [3,4]. A key contributor to this stress is the inherently competitive nature of medical education, where students often compare their academic performance and clinical competencies with those of their peers [5,6], as their career advancement, such as admission to residency programs, depends on outperforming others. While medical training involves multiple stressors [7], peer comparison is especially important because it is both pervasive and socially embedded, shaping how students evaluate themselves in relation to others rather than in absolute terms [8]. In such environments, relative performance may become internalised as a marker of social standing among peers [9]. Students who view themselves as falling behind may experience a diminished sense of subjective social status (SSS), defined as individuals’ perceived rank relative to others and inherently shaped by social comparison processes.

Evidence shows that low SSS is consistently associated with poorer health outcomes across diverse populations [10,11]. This association is thought to arise because perceptions of lower social rank can trigger psychological and physiological stress responses that adversely affect health. From a theoretical perspective, Social Rank Theory provides a useful framework for understanding these associations [12,13]. This theory conceptualises social hierarchies as biologically and psychologically salient systems, in which perceptions of lower rank can trigger feelings of inferiority and shame, particularly when individuals compare themselves to others with higher perceived status [14]. Consistent with this framework, such negative emotions may activate the body’s threat response and trigger chronic stress and sustained increases in stress hormones such as cortisol, especially among those who perceive themselves as unable to improve their relative standing [11]. These psychosocial and biological processes help explain why individuals who view themselves as lower on the social ladder consistently report poorer physical and mental health, including increased depressive symptoms and lower overall well-being, compared to those who perceive their status as higher [11,15].

The role of peer SSS may be especially pronounced in honour-based societies, such as Saudi Arabia [16], where identity and self-worth are closely tied to social perception, family expectations, and community standing [17]. In such contexts, students may place greater emphasis on how they are perceived relative to their peers, especially in high-stakes fields like medicine, which are viewed as prestigious and socially valuable [18]. Moreover, the centrality of family prestige and socioeconomic reputation in many Middle Eastern cultures, including Saudi Arabia, further amplifies the importance of peer SSS [16]. Consequently, medical students may experience pressure not only to achieve personal success but also to uphold or enhance their family’s social standing, thereby intensifying peer comparisons. However, the present study did not directly measure honour values; cultural considerations are presented as contextual background, rather than as directly tested mechanisms.

Despite growing evidence on subjective social status and health, limited research has examined these associations in young adult populations and in non-Western contexts, including the Middle East. Although medical students represent a specific subgroup, they are embedded in highly competitive and socially stratified environments, making them a useful model for studying the health implications of perceived social rank in young adult populations. This study examines the relationship between peer SSS and both physical and mental health among undergraduate medical students in Saudi Arabia. We hypothesise that students who perceive themselves as having lower peer SSS will report poorer physical and mental health outcomes compared to those with higher peer SSS. Furthermore, we will examine whether parental socioeconomic position (SEP) acts as a confounding factor in the relationship between peer SSS and student health.

## Methods

### Study design and participants

A cross-sectional observational study on peer SSS and four health outcomes was conducted at the College of Medicine, King Saud University, Riyadh, Saudi Arabia, between January and February 2025. The target population comprised all undergraduate medical students enrolled during the 2024–2025 academic year, limited to years 1–5 of study, prior to the internship year. Eligibility criteria included being a current undergraduate medical student aged 18 years or older. Participants were recruited through invitations sent to all undergraduate medical students *via* WhatsApp messaging groups available for each student group/year. The exact number of students who received or viewed the invitation could not be verified. Based on administrative estimates, ∼1,400 undergraduate medical students in years 1–5 were eligible during the study period; therefore, the participation proportion was ∼28% (390/1400). Data were collected using a self-administered online questionnaire developed in Google Forms. The questionnaire was administered in English, the language of instruction at the College of Medicine, King Saud University. It underwent pre-testing for clarity and wording, followed by pilot testing among 10 medical students prior to data collection. All procedures were performed in compliance with relevant laws and institutional guidelines and in accordance with the principles of the Declaration of Helsinki. The study protocol was reviewed and approved by the Institutional Review Board of King Saud University (Approval No. 24/1791/IRB, November 24, 2024). At the beginning of the questionnaire, participants were presented with an information and consent page and provided informed consent electronically by indicating agreement (“I agree to participate”) before proceeding to any survey items. Participation was voluntary and no incentives were offered. The privacy rights of participants have been observed.

### Measures

#### Health outcomes

Self‑rated physical and mental health were each assessed using separate single‑item questions: “How would you rate your current physical health?” and “How would you rate your current mental health?” Response options were: very good, good, neither good nor bad, bad, and very bad. For analysis, the last three categories (neither good nor bad, bad, and very bad) were combined to indicate “less than good” physical or mental health. Single-item self-rated questions on health have been shown to be valid measures that correlate with multi-item questions [19,20].

Psychological well-being was measured using the WHO-5 Well-Being Index [21], a validated self-report tool that assesses psychological well-being over the past two weeks. The instrument includes five statements: (1) I have felt cheerful and in good spirits, (2) I have felt calm and relaxed, (3) I have felt active and vigorous, (4) I woke up feeling fresh and rested, and (5) My daily life has been filled with things that interest me. Each item was rated on a 6‑point Likert scale from 0 (at no time) to 5 (all of the time), with higher scores indicating better well‑being. Item scores were summed to yield a raw total ranging from 0 to 25. In this study, the psychological well-being scale demonstrated good reliability (Cronbach’s alpha = 0.86). In accordance with the WHO guideline, scores below 13 were classified as low psychological well‑being [21].

Life satisfaction was assessed using a question: “In general, how satisfied are you with your life?” The response options included very satisfied, slightly satisfied, slightly dissatisfied, and very dissatisfied [22]. Responses were recoded into two categories: high satisfaction (very satisfied and slightly satisfied) and low satisfaction (slightly dissatisfied and very dissatisfied).

#### Subjective social status

Peer SSS was measured using the youth version of the validated MacArthur Scale of SSS, a widely used instrument depicting a 10‑rung ladder [23]. Participants rated their perceived social standing relative to their fellow students. Students were shown a ladder and asked to imagine that it represented their college, with students at the top described as those with the most respect, highest grades, and highest standing, and students at the bottom described as those whom no one respects, whom no one wants to hang around with, and who have the worst grades. The top rung represented the highest perceived status and the bottom rung the lowest, and participants selected the rung that best reflected their perception. The precise wording of the peer SSS measure and the visual representation of the ladder are provided in Supplementary Material Figure S1. Due to the small number of responses in the lowest four rungs of the ladder, these categories were collapsed into a single category. The peer SSS was then treated as a continuous variable ranging from 1 to 7, with higher values indicating higher peer SSS. This approach retained the ordinal ranking of the original ladder, allowed interpretation of the odds ratio per one-rung increase in peer SSS, and is consistent with previous studies using youth SSS ladder measures as continuous variables [24].

#### Parental socioeconomic position

Participants were asked to estimate the total income of their household from all sources in the previous month. Responses were provided in predefined categories: SAR 5,000 or less, SAR 5,001–15,000, SAR 15,001–25,000, SAR 25,001–35,000, SAR 35,001–45,000, and SAR 45,001 or more. For analysis, household income was dichotomised into relatively higher-income households (> SAR 25,000) and lower-income categories (≤ SAR 25,000). This cut-off corresponded to the boundary between two predefined response categories and was selected to identify a higher-income group in the Saudi context. According to the 2023 Household Income and Consumption Expenditure Statistics from the General Authority for Statistics, the average monthly disposable household income was SAR 18,056 among Saudi households [25]. Therefore, SAR 25,000 represents a threshold above national average household income levels. Parental education was assessed separately for fathers and mothers, using four categories ranging from less than high school to postgraduate degree. Responses were recoded into two groups: higher education (bachelor’s degree, diploma, or postgraduate) and less than higher (high school or less). Open-ended responses regarding parental occupation were classified by the research team using the International Standard Classification of Occupations 2008 (ISCO-08). Occupational groups included armed forces personnel, managers, professionals, technicians/clerical/service/sales workers, retired individuals, and the unemployed/housewives. Reports of deceased parents were recoded as missing. Family SSS was measured separately using the MacArthur Scale of family SSS [23], where students rated their family’s standing within the broader community (see Supplementary Material Figure S2). The Family SSS scale was treated similarly to the peer SSS scale (see above). Physical activity was assessed using a single-item screening question: “As a rule, do you do at least half an hour of moderate or vigorous exercise, such as brisk walking or a sport, on five or more days of the week?” Response options were “yes” and “no”. Students who responded “yes” were classified as having high physical activity, whereas those who responded “no” were classified as having low physical activity. This single-item question has previously shown acceptable validity for identifying physically inactive adults [26].

#### Covariates

Age, sex, and parental marital status were considered as covariates in this study. Parental marital status was self-reported in response to the question, ‘What is your parents’ current marital status?’ The responses were categorised into three groups: Married, Divorced/Separated, and Widowed.

### Statistical analysis

The characteristics of undergraduate medical students were described using frequencies and percentages, or means and standard deviations. The associations between student characteristics and peer SSS were evaluated using Student’s *t*-test for two-group comparisons and one-way ANOVA for comparisons involving more than two groups. For all variables that reached (*p* < 0.05) or approached (*p* < 0.10) statistical significance in the ANOVA, post hoc pairwise comparisons were carried out to identify specific group differences. Post hoc comparisons were conducted using Tukey’s HSD test when the assumption of homogeneity of variances was met, and the Games–Howell test when this assumption was violated. Pearson’s correlation coefficient (r) was used to assess the strength and direction of the relationship between family SSS and peer SSS, using the recoded 7-level SSS variables used in the regression analyses. Sequential multiple logistic regression models were employed to estimate the associations between peer SSS and each health outcome. The assumption of linearity in the logit was assessed for peer SSS and family SSS, which were entered as continuous 7-level variables, using Box–Tidwell interaction terms between each SSS variable and its natural logarithm. There was no evidence that the linearity-in-the-logit assumption was violated for peer SSS or family SSS. Age and sex were included in all regression models a priori. Additional covariates were selected based on their conceptual relevance as indicators of parental socioeconomic position or family background and their association with peer SSS in bivariate analyses at *p* < 0.10. Accordingly, monthly household income, father’s occupation, family SSS, and parental marital status were included in the sequential multivariable models. Model 1 adjusted for age and sex. Model 2 additionally adjusted for monthly household income, father’s occupation, and family SSS. Parental marital status was added to Model 3. In a sensitivity analysis, potential two-way interactions between peer SSS and age, sex, or parental SEP (monthly household income, parental education and occupation, family SSS) were examined by separately adding product terms to the logistic regression models. A complete case analysis was performed. Odds ratios (ORs) and 95% confidence intervals (CIs) were reported for all models. Variables were tested for multicollinearity using variance inflation factors (VIF). Model calibration was assessed using the Hosmer–Lemeshow goodness-of-fit test for the fully adjusted logistic regression models. Statistical significance was set at *p* < 0.05. Analyses were performed using IBM SPSS Statistics for Windows, Version 30.0 (IBM Corp., Armonk, NY, USA).

## Results

The demographic characteristics of the sample are presented in Table 1. A total of 390 undergraduate medical students from King Saud University in Riyadh, Saudi Arabia, participated in this study. The prevalence of less than good physical health was 31.4% (122/388), less than good mental health was 34.9% (136/388), low psychological well-being was 52.8% (200/379), and low life satisfaction was 19.7% (77/390). The majority of students were between 20 and 23 years of age (83.3%), male (62.3%), and single (97.4%). Most participants reported living with their families (93.8%) and had a monthly household income exceeding SAR 25,000 (approximately USD 6,667) (67.8%). Additionally, the majority reported that at least one parent had a higher education, with paternal education levels slightly higher than maternal (82.7% vs 74.3%). Paternal occupations were predominantly in professional (41.9%) and managerial roles (15.3%). Mothers were more frequently reported as professionals (44.7%) or housewives (40.7%).

**Table 1.** Means and standard deviations of peer subjective social status score by student characteristics for undergraduate medical students at King Saud University in Riyadh, Saudi Arabia, 2025 (*N* = 390).

Characteristics	*N*	%	Peer SSS score (mean, SD)	*P*-value
Age				
18–19	35	9.0	4.57 (1.63)	0.608
20–21	149	38.2	4.59 (1.79)	
22–23	176	45.1	4.48 (1.74)	
24 and older	30	7.7	4.30 (1.60)	
Sex				
Male	243	62.3	4.50 (1.64)	0.421
Female	147	37.7	4.65 (1.89)	
Marital status				
Married	10	2.6	5.00 (2.16)	0.415
Single	378	97.4	4.54 (1.73)	
Academic year				
Basic science years (1–2)	80	20.5	4.41 (1.62)	0.408
Clinical years (3–5)	310	79.5	4.59 (1.77)	
Monthly household income				
>SAR 25,000	232	67.8	4.79 (1.59)	**<0.001**
≤SAR 25,000	110	32.24	4.06 (1.91)	
Father’s education				
Higher education	311	82.7	4.53 (1.74)	0.869
High school or less	65	17.3	4.49 (1.72)	
Mother’s education				
Higher education	277	74.3	4.51 (1.73)	0.455
High school or less	96	25.7	4.67 (1.81)	
Father’s occupation				
Armed forces occupations	44	12.2	4.58 (1.97)	0.094
Managers	55	15.3	5.11 (1.51)	
Professionals	151	41.9	4.57 (1.69)	
Technicians and associate professionals, clerical support workers, service and sales workers	33	9.2	4.03 (1.83)	
Retired	77	21.4	4.56 (1.79)	
Mother’s occupation				
Managers	13	3.5	5.31 (1.49)	0.306
Professionals	168	44.7	4.43 (1.76)	
Technicians and associate professionals, clerical support workers, service and sales workers	14	3.7	4.85 (1.63)	
Retired	28	7.4	4.43 (1.50)	
Housewives	153	40.7	4.67 (1.79)	
Family SSS, mean (SD)	4.22 (1.55)		*r* = 0.473	**<0.001**
Parental marital status				
Married	337	87.5	4.62 (1.72)	0.095
Divorced/separated/widowed	48	12.5	4.17 (1.84)	
Residence type				
Villa	322	85.0	4.61 (1.69)	0.539
Apartment	39	10.3	4.42 (1.93)	
Dormitory	18	4.7	4.06 (2.19)	
Living arrangement				
With family	364	93.8	4.58 (1.72)	0.459
Away from home	24	6.2	4.30 (1.99)	
Family residence				
Riyadh	371	95.6	4.58 (1.72)	0.459
Other	17	4.4	4.30 (1.99)	
Physical activity				
High	133	34.5	4.58 (1.61)	0.830
Low	252	65.5	4.54 (1.81)	
BMI in kg/m^2^				
<25	228	58.8	4.68 (1.76)	0.159
≥25	160	41.2	4.42 (1.69)	
Chronic illness				
No	350	91.1	4.60 (1.69)	0.151
Yes	34	8.9	4.15 (2.09)	

SSS, subjective social status; SD, standard deviation; SAR, Saudi Arabian Riyals (USD 1 ≈ SAR 3.75).

Note: Due to missing values, not all numbers sum up to 390.

Bold values denote statistical significance (*p* < 0.05).

In bivariate analyses (Table 1), peer SSS varied across different family background characteristics. Students from households with a monthly income of SAR 25,000 or less reported significantly lower peer SSS compared to those with a monthly household income greater than SAR 25,000 (mean peer SSS score = 4.06 vs 4.79; *p* < 0.001). Post hoc pairwise comparisons showed (Supplementary Material Table S1) that students whose fathers were employed in technical, clerical, or service occupations had lower peer SSS scores than those whose fathers worked as managers, although this comparison was marginally significant (mean = 4.03 vs 5.11; *p* = 0.052). A moderate positive correlation was observed between family SSS and peer SSS (*r* = 0.473, *p* < 0.001). This corresponds to ∼22% shared variance (*r*^2^ = 0.224), suggesting that peer SSS and family SSS are related but distinct constructs. Finally, post hoc pairwise comparisons using the original three-category parental marital status variable showed (Supplementary Material Table S2) that students with widowed parents scored significantly lower (3.17 vs. 4.62; *p* = 0.012) than those with married parents.

**Table 2.** Odds ratios (95% confidence intervals) of poor health outcomes by one-rung increase in peer subjective social status for undergraduate medical students at King Saud University in Riyadh, Saudi Arabia, 2025.

		Less than good physical health		Less than good mental health		Low psychological well-being		Low life satisfaction	
Models	Covariates included	N/events	OR (95% CI)	N/events	OR (95% CI)	N/events	OR (95% CI)	N/events	OR (95% CI)
Model 1	+ age, sex	378/117	**0.84 (0.74–0.95)**	377/129	**0.74 (0.65–0.84)**	370/191	**0.80 (0.70–0.91)**	378/70	**0.74 (0.64–0.86)**
Model 2	+ monthly household income, father’s occupation, family SSS	310/96	0.86 (0.74–1.01)	310/107	**0.81 (0.69–0.96)**	306/158	**0.80 (0.69–0.94)**	310/59	**0.68 (0.56–0.83)**
Model 3	+ parental marital status	307 /95	**0.85 (0.72–1.00)**	307/105	**0.81 (0.68–0.95)**	303/156	**0.81 (0.69–0.95)**	307/57	**0.67 (0.54–0.82)**

SSS, subjective social status; Bold values denote statistical significance (*p* < 0.05).

In the fully adjusted model (Model 3, Table 2), each one-rung increase in peer SSS scale (one-rung increase on the MacArthur ladder) was associated with a 15–33% lower odds of having poor health outcomes, such as less than good physical (odds ratio [OR] = 0.85, 95% confidence interval [CI] = 0.72–1.00), less than good mental health (OR = 0.81, 95% CI = 0.68–0.95), low psychological well-being (OR = 0.81, 95% CI = 0.69–0.95), and low life satisfaction (OR = 0.67, 95% CI = 0.54–0.82). VIF values ranged from 1.04 to 3.76 across predictors in the fully adjusted models. The VIF values for family SSS and peer SSS were 1.45 and 1.31, respectively, indicating that multicollinearity was not a concern. Hosmer–Lemeshow goodness-of-fit tests did not indicate lack of fit for the fully adjusted models for less than good physical health (*χ*^2^ = 7.92, df = 8, *p* = 0.441), less than good mental health (*χ*^2^ = 6.23, df = 8, *p* = 0.621), or low psychological well-being (*χ*^2^ = 8.41, df = 8, *p* = 0.395). For low life satisfaction, the Hosmer–Lemeshow test was statistically significant, though borderline (*χ*^2^ = 15.84, df = 8, *p* = 0.045), suggesting possible model miscalibration; therefore, this result should be interpreted with some caution.

In a sensitivity analysis, no statistically significant interactions (*p* > 0.05) were found between peer SSS and age, sex, or parental SEP (monthly household income, parental education and occupation, family SSS) in relation to the health outcomes, suggesting that the associations did not differ meaningfully across these sociodemographic groups.

## Discussion

In this cross-sectional study of undergraduate medical students at King Saud University in Riyadh, Saudi Arabia, lower perceived peer SSS was consistently associated with poorer physical and mental health outcomes, such as greater odds of less than good physical and mental health, low psychological well-being, and low life satisfaction. The associations remained robust after adjustment for parental SEP and parental marital status, indicating that status perceptions within the student peer context capture a psychosocial dimension of student well-being not explained by family background. While SSS has been widely studied in relation to adult health outcomes, far less is known about its role during youth, and particularly among medical students, who often face unique academic, social, and psychological pressures that may shape their perceived social standing and future health and career trajectories. These findings extend beyond the context of medical education and contribute to a broader understanding of how perceived social position within peer hierarchies may shape health outcomes in young adult populations.

Our findings are consistent with the social rank theory [12,13] and align with a growing body of evidence that peer SSS is an independent factor associated with physical and mental health among students [27–29]. In competitive academic environments, daily social comparisons with classmates may amplify perceived rank differences, triggering cognitive-affective processes such as inferiority, shame, and threat appraisal. These processes are consistent with established pathways linking social hierarchy to health through chronic stress activation and psychophysiological dysregulation. They are also well-documented contributors to anxiety, depressive symptoms, and diminished well-being [30,31]. The prominence of peer SSS in medical training is particularly noteworthy, as performance is frequently evaluated, publicly visible, and directly tied to future career opportunities such as residency placement. However, the reverse direction is also plausible: students with poorer mental health, or lower well-being, may perceive their peer status more negatively. Longitudinal studies are therefore needed to clarify whether lower peer SSS precedes poorer health.

Although our data cannot directly compare honour‑based with other cultural contexts, one possible contextual explanation is that peer SSS may be especially salient in honour-based societies such as Saudi Arabia [16]. Unlike dignity cultures, where self-worth is viewed as intrinsic, or face cultures, where harmony and collective reputation are prioritised, honour cultures place particular emphasis on individual achievements as reflections of family and community standing [17]. In this context, medical education may represent not only a personal career trajectory but also a socially prestigious arena in which success or failure may carry reputational consequences. Thus, peer comparisons may extend beyond academic competition to encompass broader evaluations of honour and respect, potentially amplifying the psychosocial burden of perceived low status. However, the present study did not measure honour values, family prestige, or cultural norms directly. Therefore, the possibility that cultural expectations intensify peer comparisons in medical education should be regarded as a contextual interpretation rather than an empirical finding of this study. Future cross-national research should investigate whether cultural factors moderate the association between peer SSS and student health.

This study contributes to the limited literature on SSS in Middle Eastern academic contexts. Methodological strengths include the use of validated instruments, examination of multiple health outcomes, and measurement of a comprehensive set of potential confounders, including parental SEP. Nonetheless, several limitations should be acknowledged. First, the cross-sectional design precludes causal inference and raises the possibility of reverse causation (e.g. students with poorer mental health perceiving lower peer status). Second, recruitment through WhatsApp groups may have introduced selection bias, potentially over-representing more engaged or socially connected students. Third, students may have been reluctant to report low peer standing or poor health, which could have introduced social desirability bias and influenced the observed associations. The direction and magnitude of any resulting bias are uncertain. Fourth, peer SSS and all health outcomes were self-reported in the same questionnaire, which may have introduced common-method bias and inflated associations, particularly if students with negative affectivity tended to report both lower status and poorer health. Fifth, household income was measured using predefined categories and dichotomised to identify a relatively higher-income group. Although any single income cut-off is to some extent arbitrary, the SAR 25,000 threshold was above the national average monthly disposable household income and therefore provided a contextually informed distinction between higher-income and other households. Sixth, residual confounding remains possible because we did not measure academic performance, personality traits, social support, or pre-existing mental health conditions. Seventh, the single-institution sample limits the generalisability of our findings to other medical schools in Saudi Arabia or internationally. Nevertheless, studies conducted in well-defined populations can provide valuable insights into underlying mechanisms that may be applicable to broader population groups. Eighth, missing values accounted for <5% for most variables, except for monthly household income (12.3%) and father’s occupation (7.7%). Additionally, comparisons of health outcomes between participants with and without missing data revealed no substantial differences, suggesting that bias due to differential nonresponse is unlikely. Finally, although the MacArthur youth ladder is widely used to assess subjective social status, its wording in the peer context includes several related but conceptually distinct dimensions, including academic standing, respect, social acceptance, and belonging. Because these dimensions were not measured separately, peer SSS in this study should be interpreted as a broader measure of perceived position within the student peer environment.

## Conclusions

This study shows that lower perceived social position within peer hierarchies was associated with poorer self-reported physical health, mental health, psychological well-being, and life satisfaction among undergraduate medical students in Saudi Arabia. Our findings suggest that medical schools may benefit from greater attention to the social dynamics of training as one potential factor related to student well-being. Institutional strategies, such as adjusting grading systems to reduce excessive competition and establishing small-group faculty advisor/mentor programs that foster collaborative learning environments, may represent promising areas for future evaluation [32]. Ensuring access to high-quality mental health services and evidence-based wellness programs may also be important [32], although the present study did not evaluate these interventions directly. These findings contribute to the growing body of evidence emphasising the role of relative social position in shaping health inequalities. Further longitudinal and intervention studies are particularly needed to clarify the direction of the association and evaluate whether modifying social and educational environments can improve student well-being. Cross-national studies can additionally provide valuable insights into the potential influence of cultural factors on the relationship between peer SSS and student health.

## Supplementary Material

S1 Fig.pdf

S1 Table.pdf

S2 Fig.pdf

S2 Table.pdf

## Data Availability

The data that support the findings of this study are available from the corresponding author, A.A.T., upon reasonable request.
