# Supplementary material for: Subjective social status and health of Saudi medical students: a cross-sectional study on the role of peer comparisons
Source: Ann Med. 2026 Aug 3;58(1):2708520. doi: 10.1080/07853890.2026.2708520 (PMC13435282; doi:10.1080/07853890.2026.2708520)
Supplement: Supplementary file 1 [file IANN_A_2708520_SM5415.pdf]

S2 Table. Post-hoc pairwise comparisons of peer subjective social status (SSS) by parental marital status using Tukey's honestly significant difference (HSD) test.

Parental marital status (I)	Parental marital status (J)	Mean difference (I-J)	SE	Tukey-adjusted P	95% CI lower	95% CI upper
Married	Divorced/Separated	0.115	0.303	0.923	-0.597	0.828
Married	Widowed	1.449	0.507	0.012	0.256	2.641
Divorced/Separated	Widowed	1.333	0.575	0.054	-0.019	2.686

Note: Overall ANOVA: $F(2, 370) = 4.109$, $P = 0.017$
