# Supplementary material for: Subjective social status and health of Saudi medical students: a cross-sectional study on the role of peer comparisons
Source: Ann Med. 2026 Aug 3;58(1):2708520. doi: 10.1080/07853890.2026.2708520 (PMC13435282; doi:10.1080/07853890.2026.2708520)
Supplement: Supplementary file 2 [file IANN_A_2708520_SM5416.pdf]

- Imagine that this ladder pictures how Saudi society is set up.

At the top of the ladder are the people who are the best off — those who have the most money, the highest amount of schooling, and the jobs that bring the most respect. At the bottom are people who are the worst off — those who have the least money, little or no education, no job, or jobs that no one wants or respects.

Now think about your family.

Please tell us where you think your family would be on this ladder. Choose the rung (letter) that best represents where your family would be on this ladder.

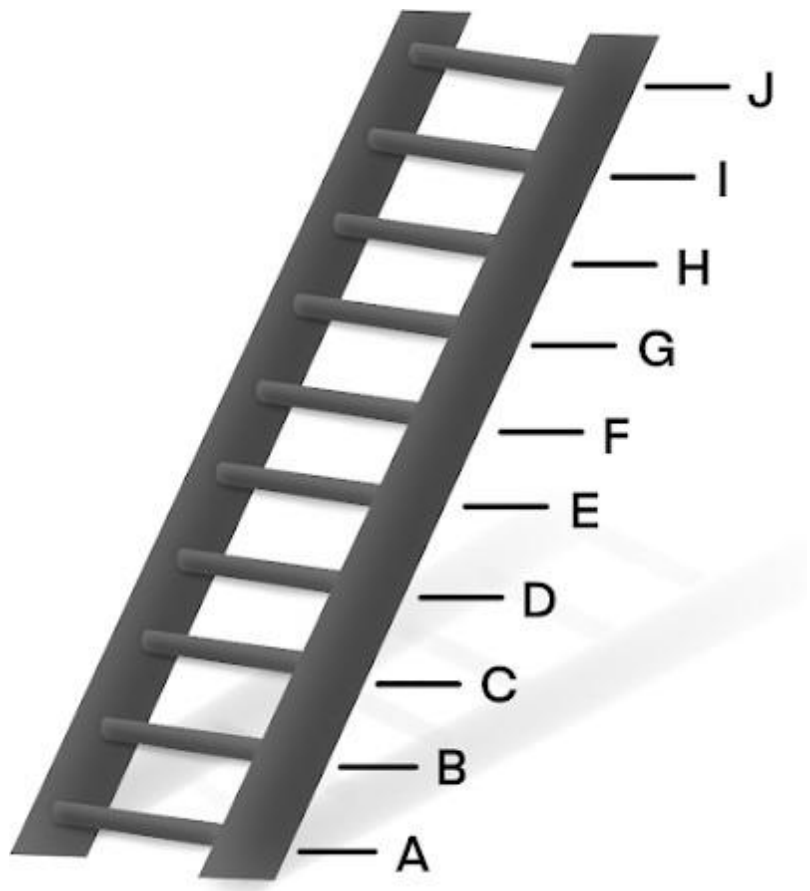

S2 Fig. Exact wording of the family subjective social status (SSS) question as presented to participants, adapted from Goodman et al. (2001).
