# Supplementary material for: Subjective social status and health of Saudi medical students: a cross-sectional study on the role of peer comparisons
Source: Ann Med. 2026 Aug 3;58(1):2708520. doi: 10.1080/07853890.2026.2708520 (PMC13435282; doi:10.1080/07853890.2026.2708520)
Supplement: Supplementary file 3 [file IANN_A_2708520_SM5417.pdf]

S1 Table. Post-hoc pairwise comparisons of peer subjective social status (SSS) by father's occupation using Tukey's honestly significant difference (HSD) test.

Father's occupation (I)	Father's occupation (J)	Mean difference (I-J)	SE	Tukey-adjusted P	95% CI lower	95% CI upper
Armed Forces Occupations	Managers	-0.530	0.355	0.567	-1.502	0.443
Armed Forces Occupations	Professionals	0.016	0.301	1.000	-0.810	0.842
Armed Forces Occupations	Technicians/clerical/service/ sales workers	0.548	0.413	0.674	-0.584	1.680
Armed Forces Occupations	Retired	0.023	0.330	1.000	-0.883	0.929
Managers	Professionals	0.546	0.277	0.282	-0.213	1.304
Managers	Technicians/clerical/service/ sales workers	1.078	0.395	0.052	-0.006	2.161
Managers	Retired	0.553	0.308	0.378	-0.292	1.397
Professionals	Technicians/clerical/service/ sales workers	0.532	0.348	0.544	-0.422	1.486
Professionals	Retired	0.007	0.245	1.000	-0.664	0.678
Technicians/clerical/service/ sales workers	Retired	-0.525	0.373	0.624	-1.549	0.499

Note: Overall ANOVA: $F(4, 344) = 1.998$, $P = 0.094$
