# Supplementary material for: Subjective social status and health of Saudi medical students: a cross-sectional study on the role of peer comparisons
Source: Ann Med. 2026 Aug 3;58(1):2708520. doi: 10.1080/07853890.2026.2708520 (PMC13435282; doi:10.1080/07853890.2026.2708520)
Supplement: Supplementary file 4 [file IANN_A_2708520_SM5418.pdf]

- Imagine that this ladder is a way of picturing your college.

At the top of the ladder are the students in your college with the most respect, the highest grades, and the highest standing. At the bottom are the students whom no one respects, whom no one wants to hang around, and who have the worst grades.

Where would you place yourself on this ladder? Choose the rung (letter) that best represents where you would be on this ladder.

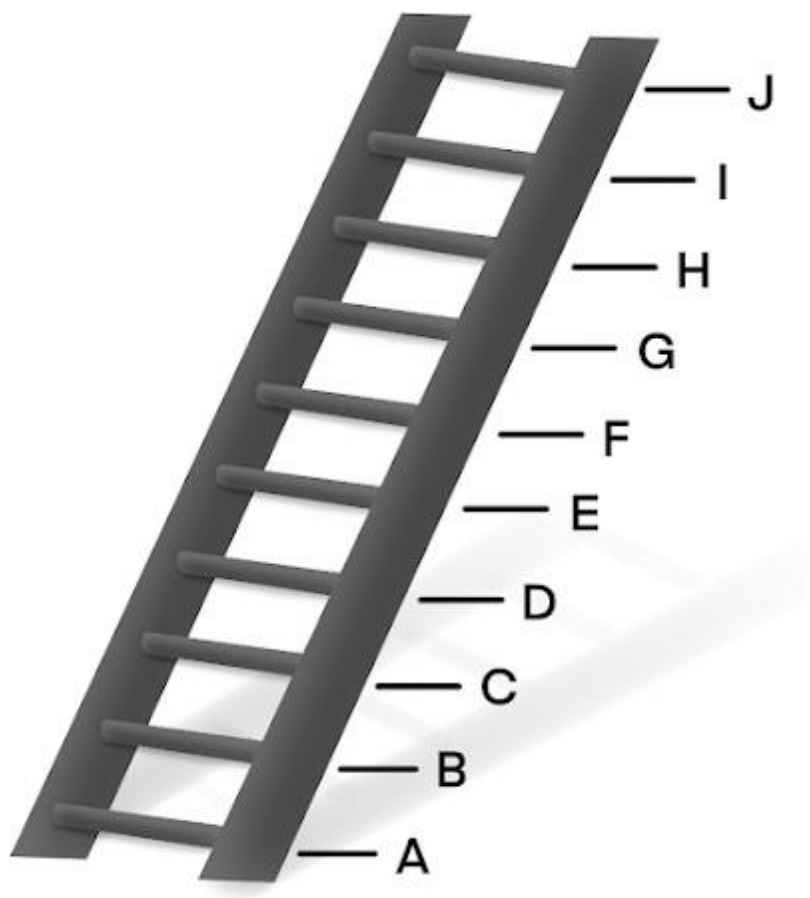

S1 Fig. Exact wording of the peer subjective social status (SSS) question as presented to participants, adapted from Goodman et al. (2001).
